# Construction of a predictive model and prognosis of left ventricular systolic dysfunction in patients with sepsis based on the diagnosis using left ventricular global longitudinal strain

**DOI:** 10.1186/s40560-022-00621-8

**Published:** 2022-06-15

**Authors:** Jiangquan Yu, Ruiqiang Zheng, Penglei Yang, Daxin Wang

**Affiliations:** 1 Medical College, Yangzhou University, Yangzhou, 225001 China; 2 Intensive Care Unit, Northern Jiangsu People’s Hospital, Yangzhou, 225001 China; 3 Intensive Care Unit, Jiangdu People’s Hospital, Yangzhou, 225299 China

**Keywords:** Sepsis, Left ventricular systolic dysfunction, Echocardiography, Predictive model, Left ventricle global longitudinal strain

## Abstract

**Background:**

Cardiac dysfunction, a common complication of sepsis, is associated with increased mortality. However, its risk factors are poorly understood, and a predictive model might help in the management of cardiac dysfunction.

**Methods:**

A monocentric prospective study of patients with sepsis was performed. Left ventricular global longitudinal strain (LV GLS) was measured using echocardiography within 72 h of the patients diagnosed with sepsis, and the patients were categorized into two groups: LV GLS > -17%, left ventricular systolic dysfunction group (LVSD group); and LV GLS ≤ -17%, non-left ventricular systolic dysfunction group (Non-LVSD group). The baseline characteristics and prognosis of the two groups were analyzed. Based on the results of the multivariate logistic regression analysis, a predictive model of LVSD was established and a nomogram was drawn.

**Results:**

Fifty-one left ventricular systolic dysfunction in patients with sepsis and 73 non-LVSD sepsis patients were included. Prognostic analysis showed that patients with LVSD had higher ICU mortality, in-hospital mortality, the incidence of atrial fibrillation (*P* < 0.05), and risk of death (HR = 3.104, 95% CI = 1.617–5.957, *P* < 0.001) compared to patients with non-LVSD. There were no significant differences in the rate of tracheal intubation, the incidence of acute kidney injury (AKI), the proportion of continuous renal replacement therapy (CRRT), length of ICU stay, and length of hospital stay between the 2 groups (*P* > 0.05). High sensitive troponin I (Hs-TnI) ≥ 0.131 ng/ml, procalcitonin (PCT) ≥ 40 ng/ml, lactate (Lac) ≥ 4.2 mmol/L, and N-terminal pro-brain natriuretic peptide (NT-proBNP) ≥ 3270 pg/ml were found to be the best cut-off values for the prediction of LVSD.

**Conclusion:**

Sepsis patients with left ventricular systolic dysfunction had a higher risk of death and atrial fibrillation. Hs-TnI, PCT, Lac, and NT-proBNP were independent risk factors of LVSD, and the LVSD predictive model constructed using these factors showed good diagnostic performance.

*Trial registration:* Chinese Clinical Trial Registry No: ChiCTR2000032128. Registered on 20 April 2020, http://www.chictr.org.cn/showproj.aspx?proj=52531.

## Background

Sepsis patients complicated with cardiac dysfunction have been recognized for more than 40 years [1, 2]. The mortality due to sepsis related to cardiac dysfunction (SRCD) is high, its pathogenesis is not fully understood, and the diagnostic criteria are not yet defined. Early detection of SRCD is important for prognosis and guiding treatment [3]. In previous studies, left ventricular ejection fraction (LVEF) < 50% was used as a criterion to diagnose left ventricular systolic dysfunction in patients with sepsis [4]. However, EF is significantly affected by afterload. Studies have shown that left ventricular global longitudinal strain (LV GLS) is more sensitive than LVEF in the diagnosis of left ventricular systolic dysfunction in patients with sepsis and has a higher correlation with prognosis [5]. Few predictive models of left ventricular systolic dysfunction in patients with sepsis have been proposed. In the current study, LV GLS was used to diagnose left ventricular systolic dysfunction. Using a case–control study, the related risk factors were explored to understand the prognosis of patients with left ventricular systolic dysfunction and establish a predictive model. With the current study, a portion of heart failure patients with preserved ejection fraction might be identified. Also, the difference in the prognosis between patients with left ventricular systolic dysfunction and patients with normal cardiac function was analyzed, and we hope to provide more clinical evidence for left ventricular systolic dysfunction in patients with sepsis.

## Materials and methods

### Study design and patients

This monocentric prospective study was carried out in the Northern Jiangsu People's Hospital, China, between April 2020 and January 2021. Inclusion criteria: (1) adult patients > 18 years old; (2) meeting the criteria for sepsis-3 from the *Surviving Sepsis Campaign: International Guidelines for Management of Sepsis and Septic Shock: 2016* [6]. Exclusion criteria: (1) severe arrhythmias (such as persistent or permanent atrial fibrillation); (2) pregnancy; (3) congenital heart disease; (4) moderate-to-severe valvular disease or history of valve replacement; (5) acute myocardial infarction or myocarditis, chronic heart failure; (6) terminal stage of cancer; (7) patients diagnosed with sepsis in other wards for more than 24 h before being transferred to the center; (8) simultaneous participation in other studies. Elimination criteria: patients who died, whose echocardiography was not performed within 72 h, and whose echocardiogram images were unclear or could not be used to measure LV GLS.

## Methods

Based on the previous results [7, 8], LV GLS > -17% was defined as left ventricular systolic dysfunction in the current study. LV GLS was measured using 2D spot echocardiography (GE Healthcare, Marlborough, MA, USA) with a frequency of 1.7–3.4 MHz, and M5s probe within 72 h of the patients diagnosed with sepsis. Chest electrocardiogram was performed in the left lateral decubitus position to obtain left ventricular end-systolic diameter, left ventricular end-diastolic volume, and left ventricular ejection fraction. These parameters were measured three consecutive times and a mean value was considered. Two-dimensional images of 3 cardiac cycles were collected from three parasternal left ventricular short-axis sections, including the left ventricular mitral valve level, papillary muscle level, and apex level, and three left ventricular long-axis sections, including apex four-chamber heart, three-chamber heart, and two-chamber heart. The images were stored and analyzed offline. Image analysis was performed using EchoPac workstation (GE Healthcare USA): clear 2D images were selected, the time points of aortic valve opening and closing were determined using the Doppler flow spectrum, and then the above three left ventricular short-axis and left ventricular long-axis section of six aspects was selected. The endocardial surface of the 6 dynamic images of the section was manually drawn. The software drew the epicardial surface automatically, and the system automatically generated the longitudinal and circumferential strains for each layer. The region of interest was manually adjusted to ensure satisfactory tracking and the global longitudinal strain (GLS) of the left ventricle was recorded. An experienced researcher from the Echocardiology department performed the ultrasound examination and obtained the images of the patients, and another researcher, blinded to the basic information of the patients, analyzed the images to ensure the accuracy of the data.

### Data collection

Demographic characteristics and underlying medical conditions were recorded systematically for each case. Clinical parameters, including sites of infection, the proportion of septic shock patients in those with sepsis, and chronic coexisting conditions were recorded. The worst value of respiratory rate, blood pressure, heart rate, and body temperature within 24 h since the diagnosis of sepsis were recorded. Vasopressor dosing intensity (VDI) was the highest value within 24 h after the diagnosis of sepsis. The initial value of central venous pressure (CVP) after admission to ICU was recorded. The 24-h fluid balance, 24-h Sequential Organ Failure Assessment (SOFA), and Acute Physiology and Chronic Health Evaluation (APACHE II) score were recorded. LV GLS value, and the worst EF value in the first 72 h after enrollment were recorded. ICU mortality, in-hospital mortality, the rate of endotracheal intubation, the incidence of atrial fibrillation, the incidence of acute kidney injury (AKI), the proportion of patient with continuous renal replacement therapy (CRRT), the length of hospital stay, and the length of ICU stay were also recorded.

### Statistical analysis

Statistical analyses were performed using the Stata software (v15.1; College Station, TX, USA). Normality tests were performed on continuous variables. Normally distributed measurement data were expressed as mean ± standard deviation. Inter-group comparisons were performed using an independent sample *t*-test. Non-normally distributed parameters were expressed as median (interquartile range) [M (IQR)]. Wilcoxon rank-sum test was used for comparison between groups and *χ*^2^ test was used for comparison of measurement data between groups. Receiver operating characteristic (ROC) curves were drawn for parameters with significant differences between the two groups to identify the optimal cut-off value, and the results were converted to dichotomous variables according to the optimal cut-off value. Factors associated with left ventricular systolic dysfunction and mortality were analyzed using multivariate conditional logistic regression models. A backward stepwise logistic regression was performed to select variables for the multivariate models, using a cut-off *p*-value of 0.1. The goodness of fit was tested using the Hosmer–Lemeshow test. The risk factors were input into the R X643.6.3 statistical software by R Studio, and the "RMS" language package was used to draw a nomogram. New scores were developed to predict the risk of left ventricular systolic dysfunction for septic patients. Bootstrap was used for internal verification to calculate the C-index, to draw the correction curve of the nomogram, and to evaluate the consistency of the results. Kaplan–Meier method was used to draw the survival curve and the log-rank test was used to calculate the hazard ratio (HR). *P* < 0.05 was considered statistically significant.

## Results

### Study population

**Fig. 1:**
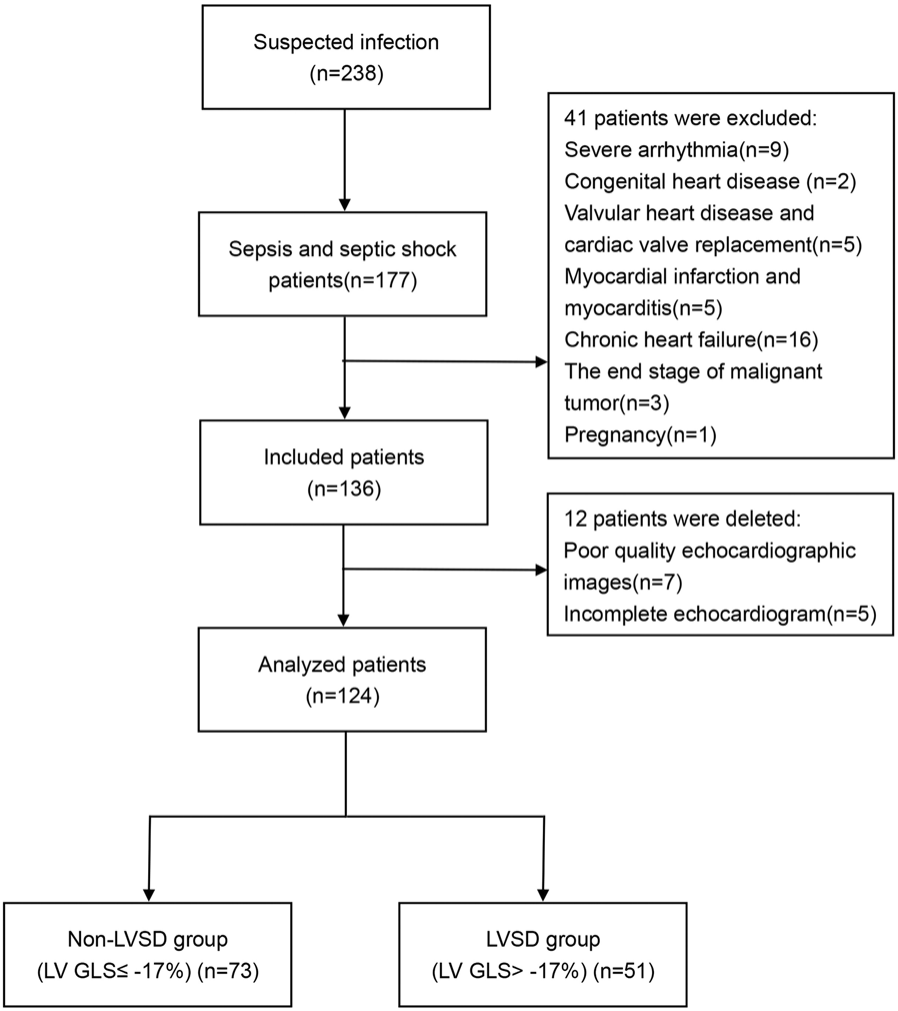
Enrollment and outcomes

From April 2020 to January 2021, a total of 177 patients were admitted to the department, of which, 41 patients were excluded (9 patients with severe arrhythmia, 1 pregnant patient, 2 patients with congenital heart disease, 5 patients with moderate-to-severe valvular heart disease or history of valve replacement, 5 patients with suspected acute myocardial infarction or myocarditis, 3 patients with end-stage cancer, and 16 patients with chronic heart failure). The remaining 136 patients were included in the study, of which, 12 patients were eliminated (5 patients did not complete echocardiography within 72 h, and the images of 7 patients were unclear and could not be analyzed using the EchoPac workstation). Therefore, data on 124 patients were finally considered for the analyses. The left ventricular systolic dysfunction group (LVSD group, LV GLS > − 17%) included 51 patients (41.1%) and the non-left ventricular systolic dysfunction group (Non-LVSD group, LV GLS ≤ − 17%) included 73 patients (58.9%) (Fig. 1).

### Baseline characteristics and prognosis of the two groups

**Fig. 2:**
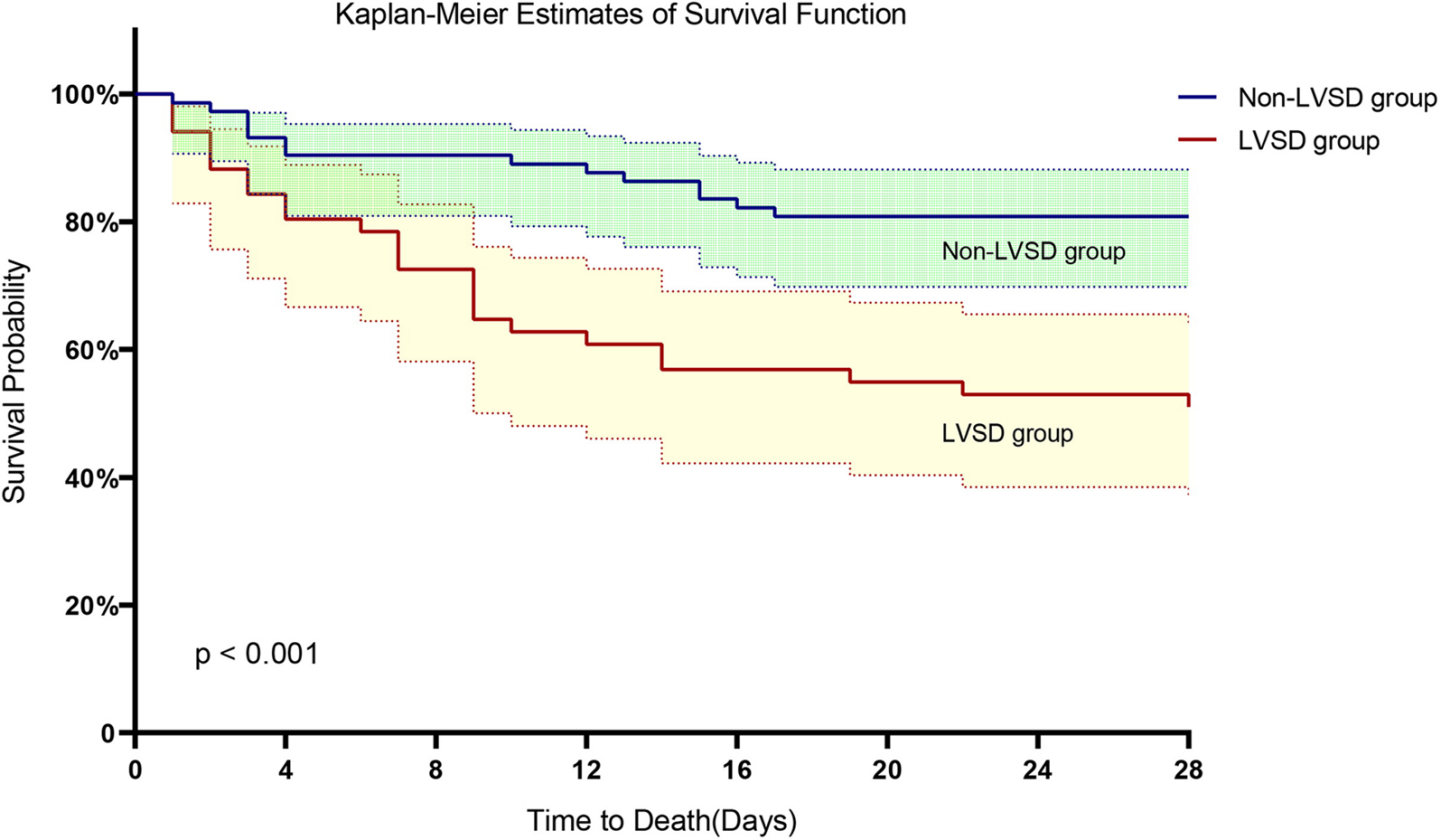
Non-LVSD group and LVSD group on 28-day survival. The Kaplan–Meier method was used to estimate the probability of survival. In the adjusted analyses, there was significant difference between the two groups with respect to death at 28 days (hazard ratio was 3.104; 95% confidence interval [CI], 1.617 to 5.957). Results have not been adjusted for multiple comparisons. The shading indicates 95% confidence intervals. LVSD: left ventricular systolic dysfunction

Comparison of baseline characteristics like age, gender, BMI, chronic coexisting conditions (hypertension, diabetes, chronic renal insufficiency, chronic respiratory failure), sites of infection, and proportion of patients with septic shock showed no significant differences between the groups (*P* > 0.05) (Table 1). There were no significant differences in the clinical parameters of respiratory rate, blood pressure, heart rate, body temperature, CVP, 24-h fluid balance, and APACHE II scores between the 2 groups (*P* > 0.05). Also, there were no significant differences in the laboratory parameters of hemoglobin, white blood cell count, platelet count, bilirubin level, serum creatinine, pH, and proportion of arteriovenous CO_2_ gap (P(V-A) CO_2_ GAP) > 6 mm Hg between the two groups (*P* > 0.05). However, Hs-TnI, PCT, Lac, NT-probNP, SOFA, and VDI were significantly higher in the LVSD group compared to the Non-LVSD group (*P* < 0.05) (Table 1). The ICU mortality, hospital mortality, and proportion of atrial fibrillation in the LVSD group were significantly higher than those in the Non-LVSD group (*P* < 0.05) (Table 2). There were no significant differences in the proportion of tracheal intubation, AKI, and CRRT, length of ICU stay, and length of hospital stay between the two groups (*P* > 0.05) (Table 2). There was no significant differences in EF between survivors and non-survivors [58% (54%, 60%) vs 55% (50%, 59%), *P* = 0.060], but non-survivor patients had higher LV GLS [17.6% (16.8%, 18.6%) vs 16.8% (15.4%, 17.8%), *P* = 0.007]. Kaplan–Meier survival analysis results showed that the 28-day mortality of patients with LVSD was significantly higher than that of Non-LVSD patients (HR = 3.104, 95% CI = 1.617–5.957, *P* < 0.001) (Fig. 2).

**Table 1:** Demographic and clinical characteristics of patients with left ventricular systolic dysfunction and non-left ventricular systolic dysfunction

	Non-LVSD group (*n* = 73)	LVSD group (*n* = 51)	*p*
*Basic characteristic*			
Male sex—no. (%)	49 (67.1%)	37 (72.5%)	0.519
Age—year	68 (63,74)	68 (57,77)	0.931
BMI (kg/m^2^)	24.1 (21.6,26.1)	22.5 (20.1,25.2)	0.061
LV GLS (%)	17.6 (18.2,19.6)	15.9 (14.7,16.8)	< 0.001^*^
LV EF (%)	52.1 ± 0.5	51.5 ± 0.8	0.057
Septic shock—no. (%)	54 (74.0%)	39(76.5%)	0.752
*Underlying medical conditions*			
Hypertension—no. (%)	33 (45.2%)	25 (49.0%)	0.675
Diabetes—no. (%)	14 (19.2%)	8 (15.7%)	0.616
Chronic kidney injury—no. (%)	7 (9.6%)	4 (7.8%)	0.737
*Clinical characteristics*			
Respiratory rate (bpm)	24 (20,29)	22 (20,26)	0.337
Blood pressure (mmHg)	67.3 (60.7,80)	73 (63.3,85.7)	0.217
Heart rate (bpm)	110.3 ± 24.0	112.0 ± 25.5	0.714
Body temperature (℃)	37.8 ± 0.1	37.6 ± 0.1	0.476
CVP (mmHg)	8.4 ± 3.8	9.9 ± 4.9	0.087
Organ function score			
APACHE II score	16.0 (12,20)	17 (14,22)	0.352
SOFA score	9.6 ± 3.0	10.9 ± 3.4	0.027^*^
*Laboratory data*			
Leucocyte count (10^9^/L)	11.2 (6.2,19.2)	9.9 (6.0,17.5)	0.575
Hemoglobin (g/L)	108.0 ± 27.1	105.3 ± 35.9	0.853
Platelet (10^9^/L)	160 (104,215)	134 (89,224)	0.369
Albumin (g/L)	27.6(23.7,31.4)	29.7(26.232.8)	0.188
Bilirubin (mmol/L)	20.2 (12.5,30.9)	18.8 (13.4,25.8)	0.455
Plasma creatinine (umol/L)	90.3(62.8,173.5)	110.2(65.1,178.9)	0.542
PCT (ng/mL)	3.3 (0.5,21)	22 (0.7,91)	0.013
Hs-TnI (ng/mL)	0.04 (0.02,0.12)	0.27 (0.07,0.86)	< 0.001
NT-proBNP (pg/mL)	1020 (321,3060)	4560 (1740,9580)	< 0.001
pH	7.34 ± 0.09	7.32 ± 0.11	0.337
Lac (mmol/L)	2.5 (1.7, 3.8)	3.7 (2.2, 5.9)	0.008
P(v-a)CO_2_ > 6 mmHg no. (%)	27 (61.4%)	22 (64.7%)	0.762
24-h fluid balance (mL)	1994.3 ± 159.6	1892.1 ± 237.9	0.711
*Vasoactive drugs*			
VDI (ug/min)	18 (0,42)	36 (12,60)	0.007^*^
Inotropic drugs used—no. (%)	2 (2.8%)	4 (7.8%)	0.193
*Site of infection—no. (%)*			
Lungs	20 (27.4%)	20 (39.2%)	0.196
Blood	1 (1.4%)	2 (3.9%)	
Abdomen	45 (61.6%)	21 (41.2%)	
Soft tissue	2 (2.7%)	3 (5.9%)	
Urinary tract	2 (2.7%)	2 (3.9%)	
Central nervous system	2 (2.7%)	0	
Others	1 (1.4%)	3 (5.9%)	

*LVSD* left ventricular systolic dysfunction, *BMI* body mass index, *LV GLS* left ventricular global longitudinal strain, *LV EF* left ventricular ejection fraction, *SOFA* Sequential Organ Failure Assessment, *APACHE* Acute Physiology and Chronic Health Evaluation, *PCT* procalcitonin, *Hs-TnI* high sensitive troponin I, *NT-proBNP* N-terminal pro-brain natriuretic peptide, *Lac* lactate, *CVP* central venous pressure, *P(v-a)CO*_*2*_ venous-to-arterial carbon dioxide partial pressure difference, *VDI* vasopressor dosing intensity = [norepinephrine (ug/min)] + [dopamine (ug/kg/min) /2] + [epinephrine (ug/min)] + [phenylephrine (ug/min) /10] + [血vasopressin (0.01 units/min) × 2].*p < 0.05

**Table 2:** Clinical outcomes of patients with left ventricular systolic dysfunction and non-left ventricular systolic dysfunction

Outcomes	Non-LVSD group (*n* = 73)	LVSD group (*n* = 51)	p
In-ICU mortality	12(16.4%)	24(47.0%)	< 0.001
In-hospital mortality	14(19.2%)	25(49.0%)	< 0.001
Atrial fibrillation—no. (%)	6 (8.2%)	11(20.8%)	0.033
Tracheal intubation—no. (%)	53(72.6%)	36(70.6%)	0.806
AKI—no. (%)	27(37.0%)	26(51%)	0.121
CRRT—no. (%)	21(28.8%)	18(35.3%)	0.441
Median length of stay in ICU—days	7(4,14)	9(4,14)	0.811
Median length of stay in hospital—days	15(10,23)	14 (7,18)	0.094

*LVSD* left ventricular systolic dysfunction, *AKI* acute kidney injury, *CRRT* continuous renal replacement therapy

### Univariate analysis of risk factors for patients with LVSD

**Fig. 3:**
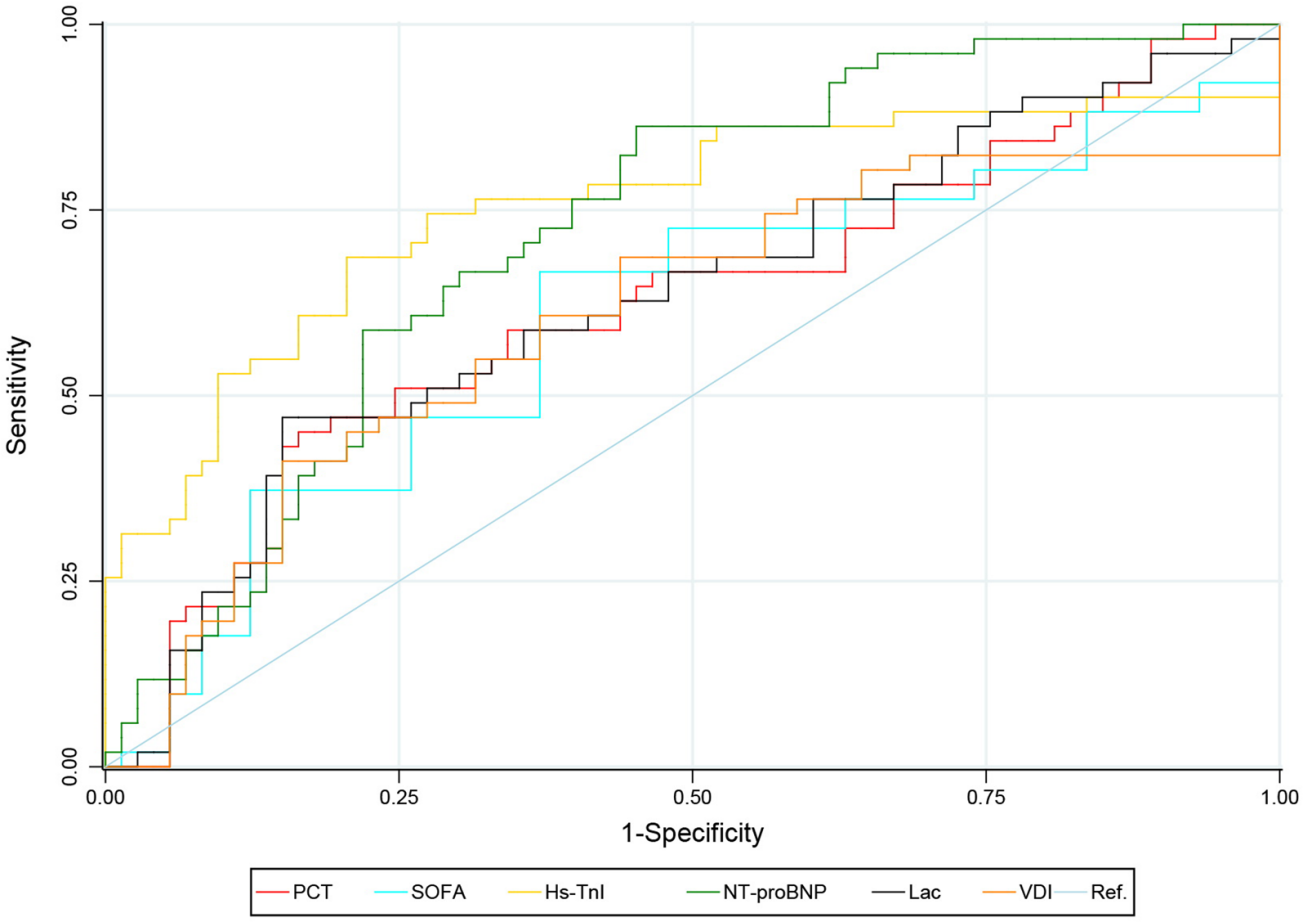
Receiver operating characteristic curve for diagnosis of left ventricular systolic dysfunction in patients with sepsis. *PCT* procalcitonin, *Hs-TnI* high sensitive troponin I, *NT-proBNP* N-terminal pro-brain natriuretic peptide, *Lac* actate, *SOFA* Sequential Organ Failure Assessment, *VDI* vasopressor dosing intensity

The diagnostic values of PCT, Hs-TnI, NT-probNP, Lac, SOFA, and VDI for LVSD (Fig. 3) were compared. The area under the curve (AUC) of PCT in the diagnosis of LVSD was 0.631, the optimal cut-off value was 40 ng/ mL, the sensitivity was 45.10%, the specificity was 83.56%, and the Youden index was 0.287. The AUC of Hs-TnI in the diagnosis of LVSD was 0.765, the optimal cut-off value was 0.131 ng/ml, the sensitivity was 68.63%, the specificity was 79.45%, and the Youden index was 0.481. The AUC of NT-proBNP in the diagnosis of LVSD was 0.726, the optimal cut-off value was 3270 pg/ mL, the sensitivity was 58.8%, the specificity was 78.1%, and the Youden index was 0.369. The AUC of Lac in the diagnosis of LVSD was 0.641, the optimal cut-off value was 4.2 mmol/L, the sensitivity was 47.1%, the specificity was 84.9%, and the Youden index was 0.320. The AUC of SOFA in the diagnosis of LVSD was 0.643, the optimal cut-off value was 11, the sensitivity was 66.7%, the specificity was 63.0%, and the Youden index was 0.297. The ADI optimal cut-off value was 57 μg/min, the sensitivity was 48.1%, the specificity was 84.9%, and the Youden index was 0.261 (Table 3).

**Table 3:** Diagnostic performance of PCT, Hs-TnI, NT-proBNP, Lac and SOFA for left ventricular systolic dysfunction in patients with sepsis

Variable	AUC	Optimal cut-off value	Sensitivity (%)	Specificity (%)	95% CI		Yoden index	p
					Upper limit	Lower limit		
PCT	0.631	40	45.1	83.6	0.528	0.733	0.287	0.013
Hs-TnI	0.765	0.131	68.6	79.5	0.675	0.856	0.481	< 0.001
NT-proBNP	0.726	3270	58.8	78.1	0.637	0.815	0.369	< 0.001
Lac	0.641	4.2	47.1	84.9	0.540	0.741	0.320	0.006
SOFA	0.643	11	66.7	63.0	0.541	0.746	0.297	0.006
VDI	0.641	57	41.2	84.9	0.541	0.741	0.261	0.006

*PCT* rocalcitonin, *Hs-TnI* high sensitive troponin I, *NT-proBNP* N-terminal pro-brain natriuretic peptide, *Lac* lactate, *SOFA* Sequential Organ Failure Assessment, *VDI* vasopressor dosing intensity = [norepinephrine (ug/min)] + [dopamine (ug/kg/min) /2] + [epinephrine (ug/min)] + [phenylephrine (ug/min) /10] + [vasopressin (0.01 units/min) × 2]. *AUC* area under the curve, *95%CI* 95% confidence interval

### Multivariate analysis of risk factors for patients with LVSD

Multivariate logistic regression analysis showed that Hs-TnI ≥ 0.131 ng/ml (OR = 6.71, 95% CI = 2.67 ~ 16.88, *P* < 0.001), PCT ≥ 40 ng/ml (OR = 3.08, 95% CI = 1.10 ~ 8.59, *P* = 0.032), NT-probNP ≥ 3270 pg/mL (OR = 2.67, 95% CI = 1.06 ~ 6.74, *P* = 0.038), and Lac ≥ 4.2 mmol/L (OR = 2.80, 95% CI = 1.02 ~ 7.69, *P* = 0.045) were significantly different. Hs-TnI ≥ 0.131 ng/ml, PCT ≥ 40 ng/ mL, NT-probNP ≥ 3270 pg/ mL, and Lac ≥ 4.2 mmol/L were independent risk factors for LVSD (Table 4).

**Table 4:** Multivariate logistic regression analysis for predictors of patients with left ventricular systolic dysfunction

Variable	Coef	SE	*z*	*p*	OR	95%CI	
						Upper limit	Lower limit
PCT > 40 ng/ml	1.12	0.52	2.15	0.03	3.08	1.10	8.59
Hs-TnI ≥ 0.131 ng/ml	1.90	0.47	4.04	< 0.001	6.71	2.67	16.88
NT-proBNP ≥ 3270 pg/ml	0.98	0.47	2.08	0.04	2.67	1.06	6.74
Lac ≥ 4.2 mmol/L	1.03	0.51	2.00	0.05	2.80	1.02	7.69

*Coef.* regression coefficient, *SE* standard error, *OR* odds ratio, *95%CI* 95%confidence interval, *PCT* procalcitonin, *Hs-TnI* high sensitive troponin I, *NT-proBNP* N-terminal pro-brain natriuretic peptide, *Lac* lactate, *SOFA* Sequential Organ Failure Assessment

### Establishment of a predictive model for patients with LVSD

**Fig. 4:**
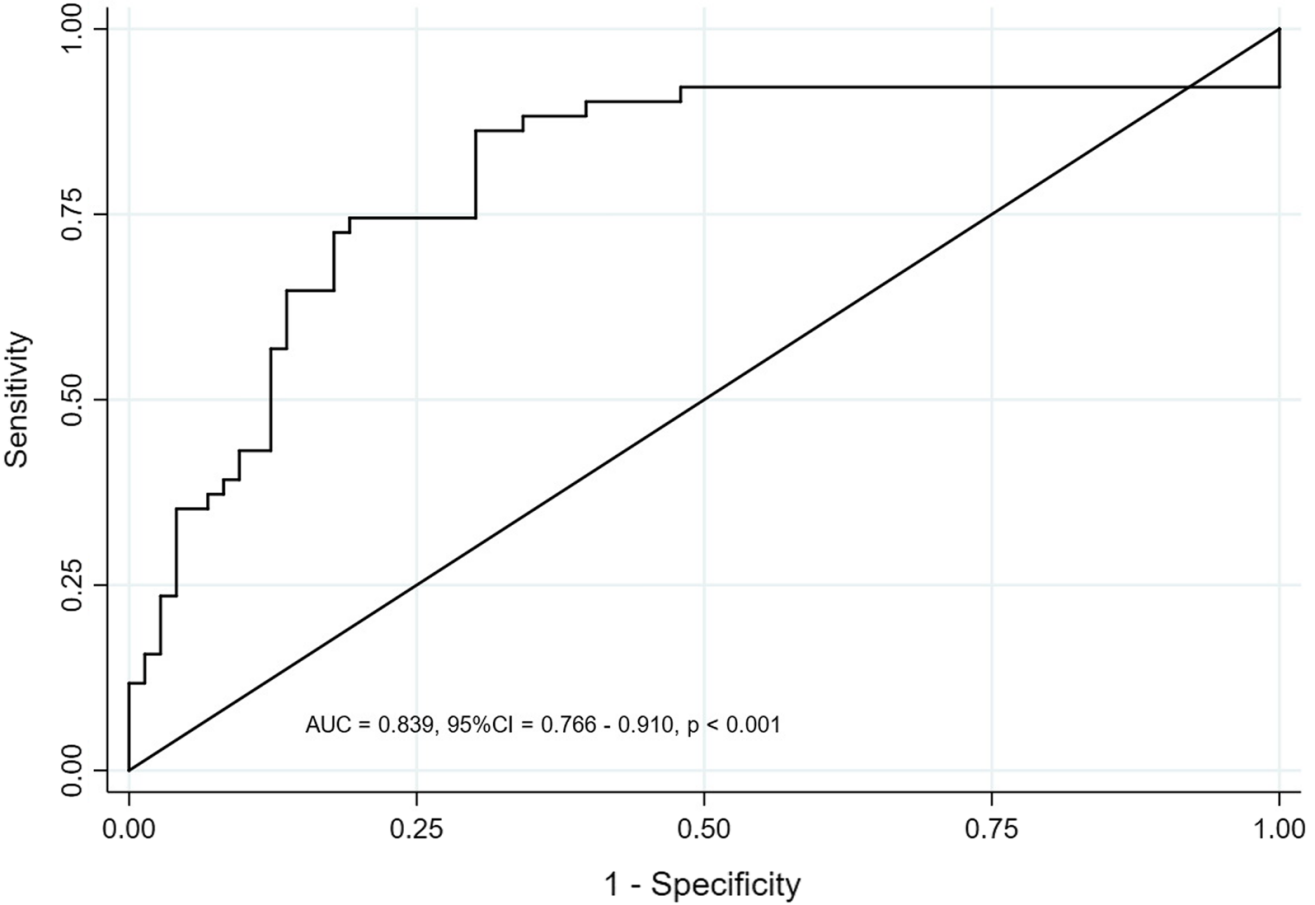
Receiver operating characteristic curves showing the predictions of the model for left ventricular systolic dysfunction in patients with sepsis

Hs-TnI ≥ 0.131 ng/ml, PCT ≥ 40 ng/ mL, NT-proBNP ≥ 3270 pg/ mL, and Lac ≥ 4.2 mmol/L were included in the logistic regression model. Logistic regression model of ln[*P*/(1-*P*)] = − 2.18 + [1.90Hs-TnI (< 0.131 ng/ml vs ≥ 0.131 ng/ml, < 0.131 ng/ml = 0, ≥ 0.131 ng/ml)] + [1.12PCT (< 40) ng/ml vs ≥ 40 ng/ml, < 40 ng/ml = 0, ≥ 40 ng/ml = 1)] + [1.03Lac (< 4.2 mmol/L vs ≥ 4.2 mmol/L, < 4.2 mmol/L = 0, ≥ 4.2 mmol/L = 1)] + [0.98Nt-proBNP (< 3270 pg/ml vs ≥ 3270 pg/ml, < 3270 pg/ml = 1)]. The Hosmer–Lemeshow test was applied and the test results showed that the model fit well (*P* = 0.719). The AUC of the predictive model was 0.839 (*P* < 0.001) (Fig. 4).

### Nomogram and verification

**Fig. 5:**
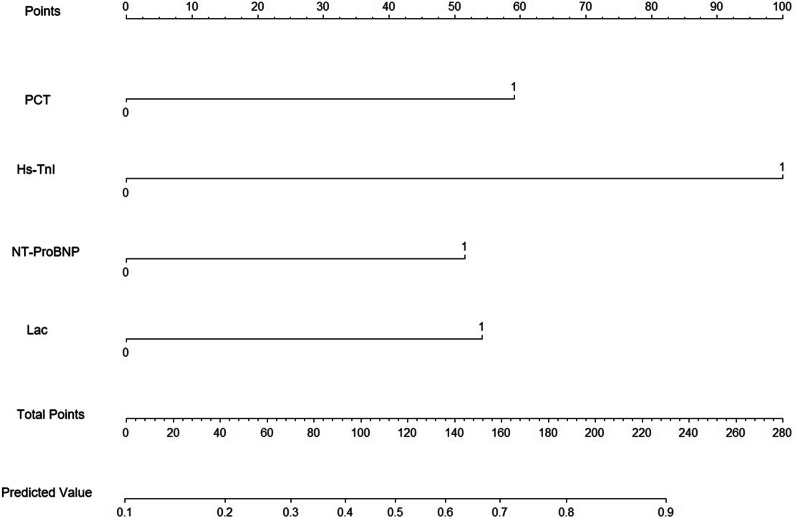
Individualized predictive nomogram model in diagnosing the left ventricular systolic dysfunction in sepsis patients. *PCT* procalcitonin, *Hs-TnI* high sensitive troponin I, *NT-proBNP* N-terminal pro-brain natriuretic peptide, *Lac* lactate

**Fig. 6:**
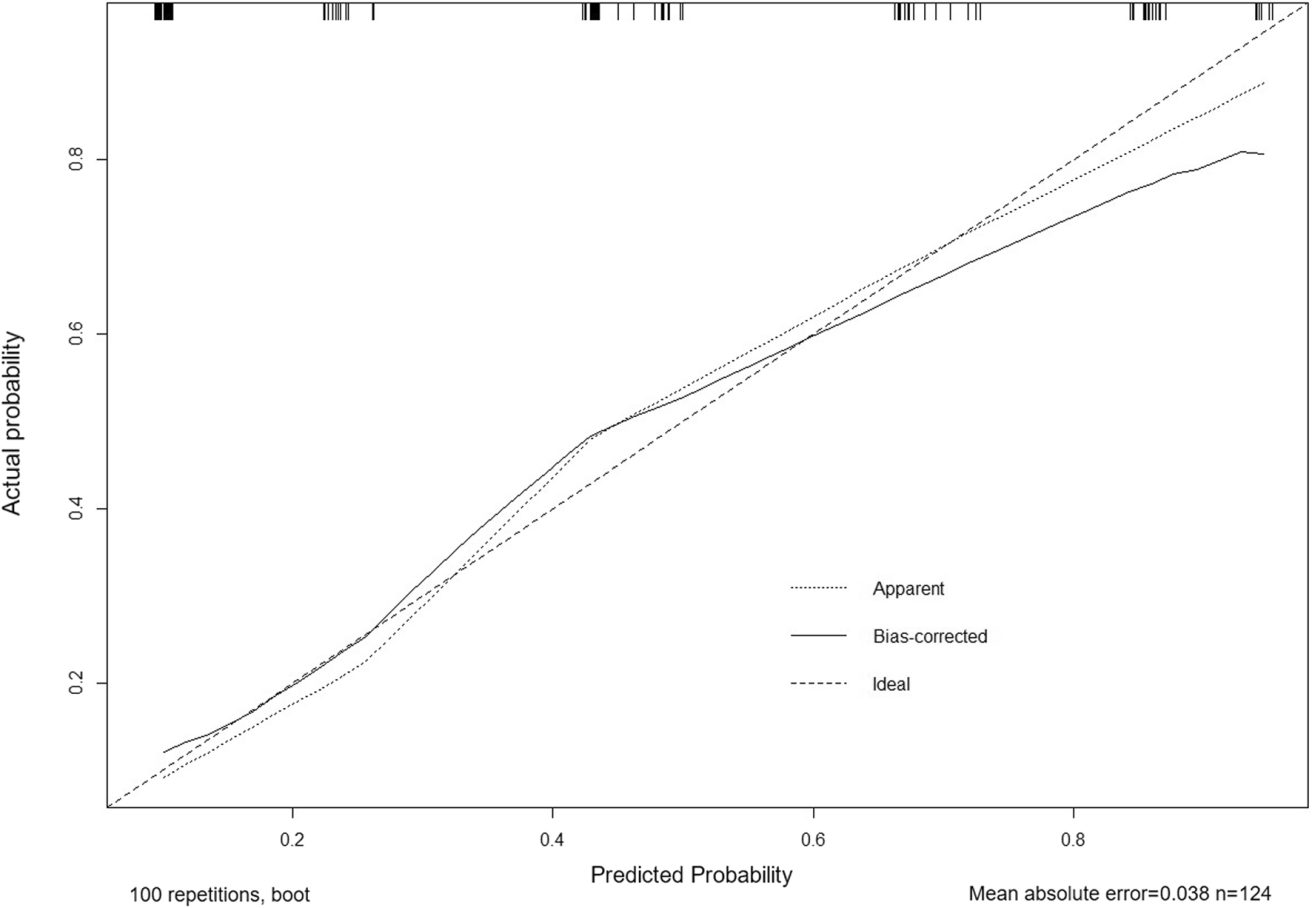
Calibration curves for the prediction of left ventricular systolic dysfunction in patients with sepsis by the nomogram

According to logistic regression results, the four risk factors: PCT ≥ 40 ng/ml, HS-TnI ≥ 0.131 ng/ml, NT-proBNP ≥ 3270 pg/mL, and Lac ≥ 4.2 mmol/L were used to draw the nomogram. The scores of the 4 factors were obtained: Hs-TnI ≥ 0.131 ng/mL, 100 points; PCT ≥ 40 ng/mL, 59 points; Lac ≥ 4.2 mmol/L, 54 points; and NT-proBNP ≥ 3270 pg/mL, 52 points (Fig. 5). The bootstrap test was used for internal verification. It was found that the correction curve was in good consistency with the ideal curve and the degree of calibration of the model was acceptable (Fig. 6). The C-index of the model was 0.822 (95% CI = 0.750–0.894).

## Discussion

Sepsis is the most common cause of death among critically ill patients. Globally, the mortality due to sepsis is about 10%, but that of septic shock can be as high as 40% [9, 10]. The heart is one of the main organs damaged due to sepsis, and sepsis patients may suffer from cardiac dysfunction due to immune disorders, oxidative stress, mitochondrial dysfunction, calcium overload, and other mechanisms [11]. A quarter of the deaths in septic shock patients are due to cardiac dysfunction [12]. Establishing an early predictive model may be helpful for the diagnosis and treatment of sepsis-related cardiac dysfunction (SRCD). More and more indicators that may be related to SRCD have been reported [13]. NT-proBNP and Hs-TnI are cardiac tissue damage biomarkers. Sepsis patients often show increased NT-proBNP and Hs-TnI, which is significantly related to the increased risk of death of patients [14, 15], but damaged heart function in sepsis is often associated with a variety of factors (the weight of each factor may be inconsistent); therefore, a comprehensive prediction model may be more valuable than a single factor. Our study showed that patients with LVSD had higher Hs-TnI, PCT, NT-proBNP, Lac, SOFA, and VDI. Moreover, Hs-TnI ≥ 0.131 ng/ml, PCT ≥ 40 ng/ml, Lac ≥ 4.2 mmol/L, NT-proBNP ≥ 3270 pg/ mL, SOFA ≥ 11, and VDI ≥ 57 μg/min had high specificity in the diagnosis of LVSD. Hs-TnI ≥ 0.131 ng/ml, PCT ≥ 40 ng/ml, Lac ≥ 4.2 mmol/L, and NT-proBNP ≥ 3270 pg/mL were independent risk factors for LVSD, and the accuracy and discrimination of the predictive model were acceptable. According to the four risk factors of LVSD in the nomogram, scores can be easily calculated, and the probability of sepsis patients complicated with cardiomyopathy can be calculated using the score.

The increase in cardiac troponin I (cTnI) is the most important warning signal for LVSD. cTnI is the only TnI isoform in human myocardial fibers and a specific marker of myocardial injury [16]. It is released early upon myocardial cell injury. As early as 2011, Røsjø et al. reported that elevated troponin levels in patients with sepsis were associated with poor prognosis [17]. Kim et al. reported that the peak concentration of Hs-TnI in septic patients with cardiac dysfunction was significantly higher than that in patients without cardiac dysfunction, and the peak concentration in the subgroup of patients with systolic dysfunction was higher than that in patients without systolic dysfunction [18]. Our study excluded 5 patients who may have acute myocardial infarction and myocarditis. It was also found that elevated Hs-TnI was significantly related to LVSD. Therefore, LVSD should be considered in patients with sepsis who show no other reason for the elevation in troponin I.

Inflammation is an important mechanism of organ damage in patients with sepsis. PCT has a good correlation with the degree of inflammation in patients [19]. Our study found that PCT in patients with LVSD is higher than that in patients with non-LVSD. PCT ≥ 40 ng/ml was an independent risk factor for LVSD, indicating that excessive inflammation may cause cardiomyocyte damage. Lactic acid is the product of tissue anaerobic glycolysis. Our study showed Lac > 4.2 mmol/L was a risk factor for LVSD. The increased Lac indicated that tissue may have suffered hypoperfusion, myocardial tissue may also have lacked perfusion. The myocardial mitochondria are damaged due to insufficient perfusion, which in turn leads to a decline in myocardial function. In addition, local metabolic acidosis may inhibit heart function directly. Innocenti et al. reported that patients with sepsis and septic shock with Lac ≥ 2 mmol/L have lower absolute values of LV GLS than patients with Lac < 2 mmol/L [20]. Our study showed similar results.

N-terminal pro-brain natriuretic peptide (NT-proBNP) is an inactive N-terminal fragment of brain natriuretic peptide (BNP). Its secretion increases with the increase in ventricular pressure and ventricular wall tension, which is an important indicator of cardiac insufficiency, and the higher the concentration, the more accurate is the diagnosis of cardiac insufficiency [21]. In 2004, Charpentier et al. reported a relationship between BNP and ejection fraction in 34 patients with sepsis and found that 15 patients with sepsis showed a decreased ejection fraction (EF < 50%), and BNP in patients with a decreased ejection fraction was significantly higher than that in patients with normal ejection fraction. Also, it was proposed that BNP can be used for the detection of cardiac function in patients with sepsis [22]. Later, it was found that the increase in BNP in patients with sepsis was correlated more strongly with the patient’s condition. Even if the ejection fraction was normal, the BNP in patients with a poor prognosis was elevated, suggesting that ejection fraction cannot sensitively and accurately reflect the cardiac dysfunction of patients with sepsis [23]. However, when LV GLS was used to diagnose left ventricular systolic dysfunction in patients with sepsis, BNP was more strongly correlated with the patient's prognosis [24]. Our study showed similar results. Patients with LVSD have higher NT-proBNP, suggesting that it can be used as one of the parameters of the predictive model for LVSD based on the diagnosis using LV GLS. However, the score of elevated NT-proBNP was the lowest among the four parameters, which may be related to the detection time. Usually, it takes more time for the compensatory secretion of BNP by the cardiomyocytes to increase after the ventricular pressure increases [25].

Our study, however, had several limitations: (1) SRCD can be categorized into left ventricular systolic and diastolic dysfunction, right ventricular systolic and diastolic dysfunction [26]. LV GLS reflects only the left ventricular systolic function, and left ventricular diastolic function and right ventricular dysfunction were not measured. An increasing number of reports suggested that sepsis-related right ventricular dysfunction is more important in SRCD and associated with the prognosis of patients. (2) GLS measurement needs to be performed by doctors specializing in ultrasound, and it is affected by different methods and analysis software. There is still no unified standard for the cut-off value of LV GLS for the diagnosis of left ventricular systolic dysfunction in patients with sepsis, and further research is needed to identify the best cut-off value. (3) NT-proBNP secretion from the heart is regulated by ventricular wall-stress, and peripheral NT-proBNP is mainly influenced by renal clearance and metabolism. In the current study, a total of 11 (8.9%) cases with history of chronic kidney disease, 53 cases (42.7%) complicated with AKI, and nearly a third of AKI patients needed CRRT. We did not rule out patients with chronic kidney disease, also not take the CRRT into account, which might affect our results. (4) The study included a small sample size, and external verification needed to be performed after the model was established.

## Conclusion

Our study showed that Hs-TnI ≥ 0.131 ng/ml, PCT ≥ 40 ng/ml, Lac ≥ 4.2 mmol/L, and NT-proBNP ≥ 3270 pg/ml are independent risk factors for left ventricular systolic dysfunction in patients with sepsis and the predictive model is acceptable. Patients with LVSD have a higher risk of death and atrial fibrillation than sepsis patients with normal cardiac function.

## Data Availability

The datasets used and/or analyzed during the current study are available from the corresponding author on reasonable request.

## Abbreviations

LV GLS: Left ventricular global longitudinal strain
LVSD: Left ventricular systolic dysfunction
SRCD: Sepsis-related cardiac dysfunction
AKI: Acute kidney injury
PCT: Procalcitonin
Lac: Lactate
NT-proBNP: N-terminal pro-brain natriuretic peptide
LVEF: Left ventricular ejection fraction
GLS: Global longitudinal strain
CVP: Central venous pressure
VDI: Vasopressor dosing intensity
SOFA: Sequential Organ Failure Assessment
APACHE II: Acute Physiology and Chronic Health Evaluation II
CRRT: Continuous renal replacement therapy
M (IQR): Median (inter-quartile range)
ROC: Receiver operating characteristic
HR: Hazard ratio
P(V-A) CO2 GAP: Arteriovenous CO2 gap
AUC: Area under the curve
CI: Confidence interval
ICU: Intensive care unit
